# Catechol cross-linked antimicrobial peptide hydrogels prevent multidrug-resistant *Acinetobacter baumannii* infection in burn wounds

**DOI:** 10.1042/BSR20190504

**Published:** 2019-06-18

**Authors:** Abidullah Khan, Miao Xu, Tengjiao Wang, Chuangang You, Xingang Wang, Haitao Ren, Hongwei Zhou, Amin Khan, Chunmao Han, Peng Li

**Affiliations:** 1 Department of Burns, Second Affiliated Hospital of Zhejiang University, School of Medicine, Jiefang Road 88, Hangzhou 310009, China; 2 Key Laboratory of Flexible Electronics (KLOFE) and Institute of Advanced Materials (IAM), Jiangsu National Synergetic Innovation Center for Advanced Materials (SICAM), Nanjing Tech University (NanjingTech), Nanjing 211816, China; 3 Shaanxi Institute of Flexible Electronics (SIFE) and Institute of Biomedical Materials and Engineering (IBME), Northwestern Polytechnical University (NPU), 127 West Youyi Road, Xi’an 710072, China; 4 Department of Clinical Microbiology, Second Affiliated Hospital of Zhejiang University, School of Medicine, Jiefang Road 88, Hangzhou 310009, China; 5 Department of Chemistry, University of Science and Technology, Bannu, Khyber Pakhtunkhwa (KPK) 28100, Pakistan

**Keywords:** Acinetobacter baumannii, Antimicrobial hydrogels, biofilm eradication, burn infection, catechol, epsilon-poly-L-lysine

## Abstract

Hospital-acquired infections are common in burn patients and are the major contributors of morbidity and mortality. Bacterial infections such as *Staphylococcus aureus* (*S. aureus*) and *Acinetobacter baumannii* (*A. baumannii*) are difficult to treat due to their biofilm formation and rapidly acquiring resistance to antibiotics. This work presents a newly developed hydrogel that has the potential for treating bacterial wound infections. The hydrogel formulation is based on an antimicrobial peptide (AMP), epsilon-poly-l-lysine (EPL) and catechol, which was cross-linked via mussel-inspired chemistry between the amine and phenol groups. *In vitro* studies showed that EPL-catechol hydrogels possess impressive antimicrobial and antibiofilm properties toward multidrug-resistant *A. baumannii* (MRAB). In addition, cytotoxicity study with the clonal mouse myoblast cell line (C2C12) revealed the good biocompatibility of this hydrogel. Furthermore, we created a second-degree burn wound on the mice dorsal skin surface followed by contamination with MRAB. Our results showed that the hydrogel significantly reduced the bacterial burden by more than four orders of magnitude in infected burn wounds. Additionally, there was no significant histological alteration with hydrogel application on mice skin. Based on these results, we concluded that EPL-catechol hydrogel is a promising future biomaterial to fight against multidrug-resistant bacterial infections.

## Introduction

Burns are a common and serious healthcare problem that require care in specialized units [1]. The nature and extent of burn injuries lead to immunosuppression in patients, resulting in increased susceptibility to various nosocomial pathogens [2]. Burn wounds act differently than traumatic wounds. Burn wounds, especially deep partial thickness and full thickness burns consist of avascular necrotic tissue (eschar), provide a protein-rich environment for bacterial colonization and proliferation [1]. Burn wounds are easily colonized by Gram-positive and Gram-negative bacteria (an average of 5–7 days) present in the hospital environment [2]. In the past few decades, Gram-negative bacteria have emerged as the most common and invasive organism by virtue of their robust antimicrobial resistance and virulence factor [3]. Additionally, biofilm formation acts as an effective barrier against antimicrobial agents and host immune cells, resulting in persistent wound infections [4].

Gram-negative bacteria, coccobacillus *Acinetobacter baumannii* is an opportunistic bacteria capable of rapidly developing drug resistance [5]. In empirical therapy, the inappropriate overuse of antibiotics led to multidrug-resistant *A. baumannii* (MRAB) outbreaks all over the world [6]. The available treatments to fight against *A. baumannii*-induced infections are toxic (e.g. colistin) and pose additional threats to patients [7]. Eradicating MRAB-associated wound bed infections is a major challenge for clinical doctors. As a consequence, antimicrobial drugs, normally antibiotics, are loaded into materials for local delivery [8–11]. However, the use of antibiotics has limitations and it increases the possibility of triggering microbe resistance [12]. For instance, *A. baumannii* strains isolated from our hospital are resistant to a wide range of antibiotics, including ciprofloxacin, gentamicin and tobramycin (Supplementary Table S1). Thus, a key challenge is to find new strategies to eliminate drug-resistant opportunistic pathogens, such as MRAB and methicillin-resistant *Staphylococcus aureus* (MRSA), from colonizing in wounds using simple, effective and low-cost treatments to develop nonantibiotic therapies.

Antimicrobial peptides (AMPs) are known as the new generation of antimicrobial molecules due to their broad-spectrum antimicrobial activity, and they are less likely to trigger drug resistance [13,14]. Cationic AMPs reduce the probability of developing drug resistance by damaging the bacterial cytoplasmic membrane [15]. However, the clinical application of AMPs is often limited because of their high cost. ε-poly-l-lysine (EPL) is a natural AMP produced by *Streptomyces albulus* [16]. It has demonstrated potent antimicrobial activity against both Gram-negative and Gram-positive bacteria such as *Escherichia coli, Pseudomonas aeruginosa, Serratia marcescens* and *S. aureus* and fungi such as *Candida albicans* [17]. Studies have shown that EPL is biodegradable, nontoxic, edible and most importantly cost effective [18]. To date, it has been widely used as a food additive, but its biomedical applications still needs further exploration [19–21].

Topical hydrogel dressings are deployed in various types of wound care [22–24]. Hydrogel provides moist environment necessary for wound bed cleaning and easy removal of necrotic tissue. The cooling effect of hydrogel reduces pain, especially in burn patients [25]. Facile-prepared hydrogels with long-lasting antimicrobial effects and minimal microbial resistance are highly valued. EPL has been employed to fabricate hydrogels with antimicrobial efficacies [17,26]. However, hydrogels preparation normally involves complex chemical synthesis, requiring organic solvents. Mussel-inspired polydopamine chemistry that occurs in the aqueous phase, is attractive method for the preparation of hydrogels [27,28]. EPL has been modified with dopamine and further cross-linked to generate a hydrogel in multistep reactions [29]. Recently, Zhao and coworkers reported that catechol could cross-link with amine-rich polymers directly to prepare thin films [30]. Catechol is less expensive than dopamine; thus, the catechol/polyamine system is an economical alternative for mussel-inspired chemistry instead of polydopamine [31].

In the present study, for the first time, we used catechol cross-linking with EPL in one step to prepare a hydrogel that demonstrates potent antimicrobial properties against MRSA and MRAB. Various types of hydrogels were synthesized based on a gradual increase in EPL concentration. The porous structure of the hydrogel was confirmed by scanning electron microscopy (SEM). *In vitro* studies were carried out to investigate the antimicrobial and antibiofilm efficacy of the hydrogel against MRAB and MRSA. A clonal mouse myoblastic cell line (C2C12) was used to evaluate the biocompatibility and cytotoxicity of the hydrogel. A second-degree mice burn model was created and infected with a clinical strain of MRAB to investigate the *in vivo* anti-infective activity of the EPL-catechol hydrogel. Furthermore, The hydrogel was applied topically and implanted subcutaneously to observe the impact on skin and biocompatibility by using the Hematoxylin and Eosin (H&E) staining technique.

## Materials and methods

### Materials

Catechol, sodium hydroxide (NaOH), tryptic soy broth (TSB), poly(ethylene glycol) diacrylate (PEGDA, Mw 700), phosphate buffered saline (PBS), Dulbecco’s modified Eagle’s solution (DMEM) and Tris/HCl were purchased from Sigma–Aldrich (U.S.A.). EPL was purchased from Bainafo Bioengineering Co. Ltd. (Zhengzhou, China). Fetal bovine serum (FBS) was purchased from Gibco (NY, U.S.A.). C2C12 cells (the clonal myoblastic cell line) were purchased from American Type Culture Collection (ATCC), (VA, U.S.A.). The LIVE/DEAD viability kits for bacteria and mammalian cells were purchased from Thermo Fisher Scientific (Waltham, U.S.A.). Luria-Bertani (LB) broth and Mueller-Hinton (MH) agar were purchased from Oxoid (Lenexa, U.S.A.). MRAB and MRSA bacterial strains were obtained from the Department of Clinical Microbiology, Second Affiliated Hospital of Zhejiang University.

### Preparation of EPL-catechol hydrogel

A series of hydrogels were prepared by mixing different ratios of EPL and catechol. Three types of hydrogels were synthesized based on the mole ratio of EPL to catechol, i.e., Gel 1: (0.3:0.1), Gel 2: (0.4:0.1) and Gel 3: (0.5:0.1) mmol/L. Briefly, EPL and catechol were dissolved in Tris/HCl solution (pH = 8.5, 10 mM). The solution was incubated at 37°C for 3 days for the partial oxidation of catecholamine. Thereafter, 500 µl of the solution was added to the 24-well plate and stored at 4°C for 2 days to make the hydrogel. PEGDA hydrogel prepared with the same concentration (wt%) prepared by UV cross-linking (Irgacure 2959, 0.1 wt%) was used as a control. All the hydrogels were washed with double distilled water for 4 h to remove the residues that did not take part in the reaction process.

### SEM of hydrogels

The morphology of hydrogels was characterized by SEM. The hydrogels were completely immersed in deionized water overnight until they reached the maximum swelling equilibrium state. They were then lyophilized by a freeze drier (Alpha 1-2LD Plus, Germany Christ) at −50°C for 48 h. The cross-section of dried samples was sputtered with gold and observed under SEM (Quanta 200, Philips-FEI, U.S.A.).

### Bacterial strain and culture conditions

Prior to antimicrobial tests, bacterial strains (MRAB, MRSA) were cultured at 37°C in TSB at 200 rpm. Overnight cultured bacteria were moved into fresh medium at a ratio of 1:100 and cultured for another 4 h to attain a log phase optical density (OD: 600 - 0.5). Cells were collected by centrifugation, washed twice with sterile PBS and then resuspended in sterile PBS at appropriate concentration of colony forming units (CFU) of bacteria (1 × 10^8^ CFU/ml). For *in vivo* studies, bacterial suspension (1 ml) was collected in sterile Eppendorf tubes, centrifuged at 4000×*g* for 4 min and washed with PBS to remove the nutrient culture medium. This process was repeated in triplicate. Finally, the testing bacteria were resuspended in PBS at a concentration of 1 × 10^8^ CFU/ml.

### Zone of inhibition study

The antimicrobial activity of EPL-catechol hydrogels was evaluated by the Kirby–Bauer (KB) test. MRAB and MRSA bacterial suspensions (1 × 10^8^ CFU/ml) were prepared as mentioned above. Then, 50 µl of bacterial suspension was transferred to MH agar on a Petri dish. Hydrogels (diameter 5 mm) with various concentrations of EPL and catechol were prepared and transferred to the dish. Sterile filter paper immersed in PBS was used as a control group. The plates were incubated at 37°C, and the zone of bacterial inhibition was measured after 24 h of incubation.

### Evaluation of *in vitro* contact antimicrobial action of EPL-catechol hydrogel

For *in vitro*, the contact antimicrobial activity of the EPL-catechol hydrogels was evaluated based on the colony count method [32]. The hydrogels were prepared in 24-well plates and rinsed with excess PBS prior to the test. MRAB bacterial suspension was prepared as mentioned above. A total of 300 µl bacterial suspension (1 × 10^8^ CFU/ml) was added to a 24-well plate containing the control group (PEGDA hydrogel) and the EPL-catechol hydrogel. After a contact time of 24 h, the samples were sonicated for 5 min to detach the adhered bacteria. Then, 50 µl aliquots were collected and plated on MH agar with ten-fold serial dilutions. The MH agar plates were incubated at 37°C for 24 h, and the number of colonies was recorded.

### SEM of MRAB seeded on hydrogel

The MRAB suspension was prepared as described above. For *in vitro* studies, 10 µl aliquots were seeded on the EPL-catechol hydrogel and control gel (PEGDA hydrogel) and incubated for 2 h. EPL-catechol hydrogel and control group (PEGDA hydrogel) were fixed in 2.5% glutaraldehyde at 4°C. The samples were fixed in 1% osmium tetroxide for 2 h and then washed with PBS and dehydrated with graded ethanol (30, 50, 70, 90 and 100%). The hydrogel samples with MRAB were sputter-coated with Pt and observed under SEM (Hitachi H-7650, Japan).

### Antibiofilm assay

Mid-log phase MRAB was harvested and resuspended in TSB with 1% glucose at a concentration of 1 × 10^8^ CFU/ml. The EPL-catechol hydrogel and tissue culture polystyrene (TCPS), used as a control group, were submerged in a 2-ml bacterial suspension for 3 days to ensure biofilm formation. The culture medium was changed every day. Specimens were washed gently with PBS to remove planktonic bacteria. The hydrogels were dried and stained with LIVE/DEAD backlight kit in a dark room following the manufacturer’s instructions. The biofilms formed on glass slides and hydrogels were observed under an inverted fluorescence microscope (IX53, Olympus, Japan). The excitation wavelengths of 488 and 561 nm were set for the detection of FITC (green channel) and Rhod (red channel), respectively. The images were analyzed using Zen 2009 software (Japan).

### *In vitro* biocompatibility assay

C2C12 cells were cultured in DMEM containing 10% heat-inactivated FBS, 1% 100 IU/ml penicillin and 100 µg/ml streptomycin at 37°C in a humidified incubator containing 5% CO_2_. Cells confluence reaching 80–90% were digested with 0.25% trypsin and passaged. The cells used in the present study were in a logarithmic growth phase and were passaged between 3 and 6. The cytotoxicity of the EPL-catechol hydrogel was investigated using the standard alamarBlue assay [33]. Prior to cell seeding, the hydrogel samples were cut into 5-mm diameter disks and placed in 70% ethanol for sterilization. C2C12 cells were seeded on a TCPS plate at a density of 6000 cells/cm^2^ for 4 h. The samples were gently placed on to the well, allowing the hydrogels to contact the cells. C2C12 cells were allowed to incubate for 1 and 4 days, and the culture medium was changed on the alternate days. After 1 and 4 days, the wells containing TCPS disks were removed. Then, 100 µl of alamarBlue reagent was added to each well and incubated for 4 h at 37°C. A total of 100 µl of the medium in each well was added to a 96-well black plate (Costar). Fluorescence was read using 530 nm as the excitation wavelength and 600 nm as the emission wavelength in a microplate reader (SpectraMax Paradigm, Molecular Devices, U.S.A.). Cells in TCPS plate wells without test materials were used as the control group. Each assay was repeated in triplicate, and the mean was calculated. The viability of C2C12 cells was examined with the LIVE/DEAD assay kit. Cultured cells at 1 and 4 days were stained with the LIVE/DEAD reagent and incubated at 37°C for 45 min. Cell adhesion and proliferation were observed with an inverted fluorescence microscope (IX53, Olympus).

### Animals

Pathogen-free BLAB/c mice (6–8 weeks of age, weighing 22–28 grams) were purchased from the Nanjing Biomedical Research Institute of Nanjing University (China). Mice had free access to food and water and were maintained on a 12-h dark/light cycle in a room with a controlled air temperature of 24 ± 2°C and humidity of 55 ± 10%. Mice were allowed to acclimatize to the new environment for a week prior to experimental studies. All animal experiments were approved and conducted under the guidance of the International Association for the Protection of Animal and Experimental Medicine and Laboratory Animal Ethics Committee of Zhejiang University, School of Medicine, Hangzhou, Zhejiang, China.

### Creation of burn wounds and MRAB infection

The dorsal surface of the mice were shaved and depilated with Veet (Reckitt Benckiser) 1 day prior to the creation of burns. Mice were divided into four groups, six mice in each group (n = 6). The following day, mice were anesthetized, and a partial thickness burn (80°C for 6 s) was created with a cylinder (diameter 1.5 cm) using a burn creation machine (ZH-YLS-5Q, Shanghai, China). Soon after burn creation, mice were resuscitated with 1 ml sterile saline. Twenty minutes later (allowing the burn wound to cool down), elastic bandage tape was applied and stripped from a 1.5 × 1.5 cm^2^ dorsal skin area eight times in one direction and eight times in the opposite direction until the skin turned red and glistened with no apparent bleeding (Supplementary Figure S1A). This method is adapted from protocols proposed by Tatiya-Aphiradee et al. [34]. Removing the upper epidermal layer provides a better environment for the attachment and incubation of bacteria (Supplementary Figure S1B). A 20-μl MRAB bacterial suspension in PBS was smeared on to the burn wound with an inoculating loop. The wound was then covered with EPL-catechol hydrogel, followed by polyurethane film (Tegaderm, 3M) application. The control group was treated with PEGDA hydrogel.

### Microbiological analysis

After 1 and 2 days of MRAB-infected burn wounds, mice were anesthetized and killed. Skin samples from control and hydrogel-treated mice were excised using a 5-mm punch biopsy (Tru-Punch, Sklar, U.S.A.). Tissue samples were then homogenized in 600 μl PBS, and ten-fold serially diluted bacterial colonies were enumerated by plating on MH agar and incubated at 37°C for 24 h. The results were normalized and expressed in log_10_ CFU bacterial load present in 1g of the tissue sample.

### SEM of MRAB infected mice skin

For *in vivo* studies, mice burn wounds were infected with MRAB followed by treatment with EPL-catechol and PEGDA hydrogel (control) for 2 days. Skin samples were collected and fixed in 2.5% glutaraldehyde and 4% paraformaldehyde followed by a wash with PBS for 30 min at 4°C. The samples were fixed in 1% osmium tetroxide for 2 h and then washed with PBS and dehydrated with graded ethanol (30, 50, 70, 90 and 100%). The samples were immersed in propylene oxide and Epon 812 for 1 h followed by polymerization embedding. Samples were cut into 1–3 µm thick sections and stained with AzurΙΙΙ or Toluidine Blue and observed under SEM (Hitachi H-7650, Japan).

### Histological analysis

Mice were divided into four groups, six mice in each group (n = 6). Following the creation of burns on the mice dorsal skin surface, the hydrogels were carefully placed on the affected skin area. Mice were anesthetized and sacrificed on days 1 and 2, and skin samples were fixed in 10% neutral buffered formalin before they were subjected to H&E staining. To evaluate the impact of the EPL-catechol hydrogel on mice skin, mice were divided into four groups, six mice in each group (n = 6), and hydrogels were implanted subcutaneously (Supplementary Figure S2A). The dorsal skin samples were collected on 2 and 5 days of implantation. All samples were processed and embedded in paraffin specimen blocks and sectioned (4-µm thick), followed by staining with H&E. To analyze inflammatory cell infiltration, H&E-stained sections were observed under a light microscope (Nikon Eclipse 80i, Japan) and photographed at ten randomly selected locations.

### Statistical analysis

Data are presented as the means ± standard deviation. Statistical analysis was executed using GraphPad Prism 5.0 (GraphPad Software, U.S.A.). *P*-values were determined using a two-tailed Student’s *t*test. *P*<0.05 was considered to be significant.

## Results

### Characterization of EPL-catechol hydrogel

**Figure 1:**
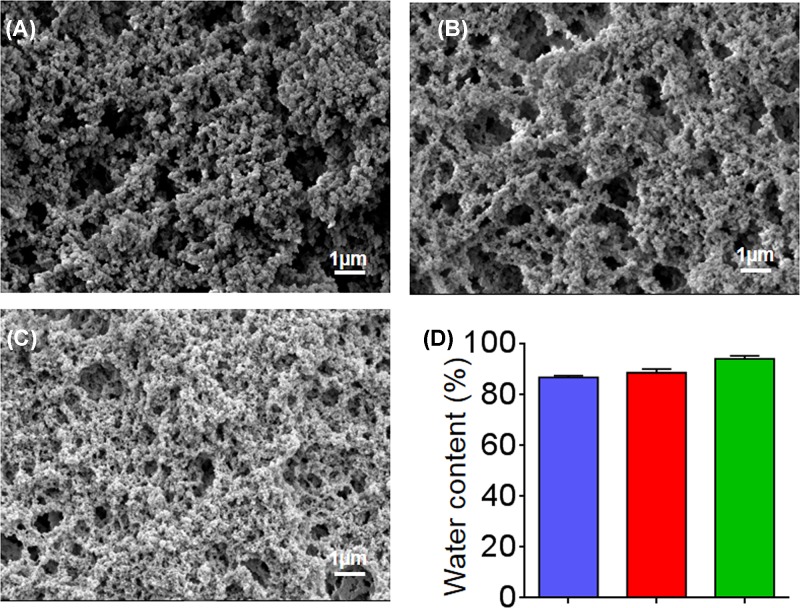
Physical characterization of EPL-catechol hydrogels SEM images of EPL-catechol hydrogels with different mole ratios of EPL to catechol: (**A**) Gel 1; 0.3:0.1, (**B**) Gel 2; 0.4:0.1 and (**C**) Gel 3; 0.5:0.1 mmol/L. (**D**) The water content of EPL-catechol hydrogels.

**Scheme 1:**
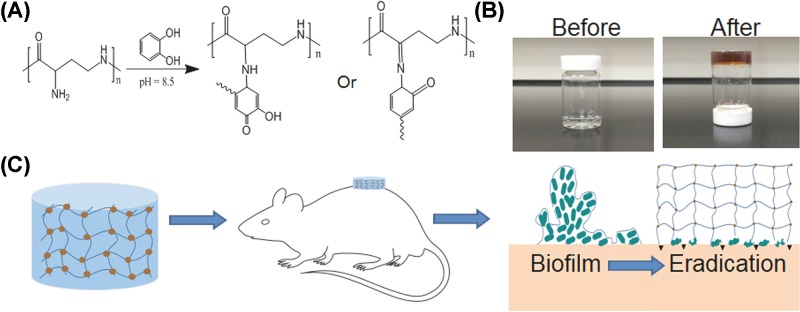
Schematic diagram of the preparation of anti-infective EPL-catechol hydrogel (**A**) The hydrogel was cross-linked by the reaction between the amine groups of EPL and the phenol groups of catechol. (**B**) The gross view of the hydrogels before (left) and after (right) gelation. (**C**) Application of EPL-catechol hydrogel as a burn wound dressing on mice.

EPL and catechol were mixed together in appropriate concentrations, and the reactions between them gradually took place. After 3 days, the transparent mixture turned into a brownish color, and the EPL-catechol hydrogels were formed without precipitation (Scheme 1 and Supplementary Figure S3A–D). The hydrogels were freeze-dried, and their microstructures were observed under SEM (Figure 1). As shown in Figure 1A–C, highly interconnected porous structures of the EPL-catechol hydrogels were observed, and the pore size decreased with increasing EPL concentration. The EPL-catechol hydrogels also exhibited high water content in the range of 87–94%. As shown in Figure 1D, the water content of the EPL-catechol hydrogels also increased as the concentration of EPL increased. There was no significant difference in water content among all three types of EPL-catechol hydrogels (Gel 1, Gel 2 and Gel 3).

### *In vitro* antimicrobial activity of EPL-catechol hydrogel

**Figure 2:**
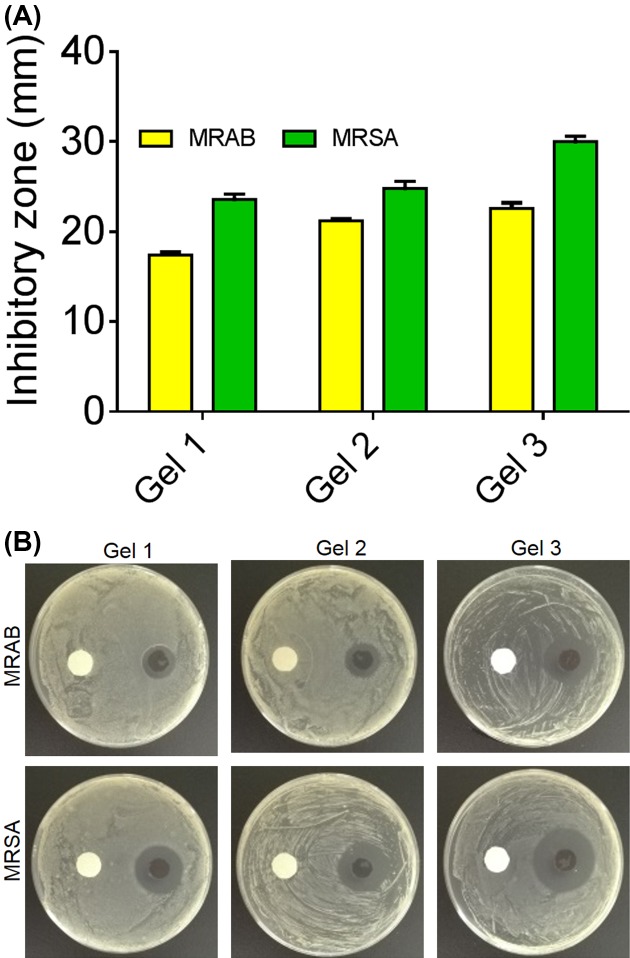
*In vitro* antimicrobial activityof EPL-catechol hydrogels determined by Kirby-Bauer (**A**) The sizes of inhibitory zones (mm) MRAB and MRSA. (**B**) images representing inhibitory zones of, Left: control (sterile disc paper) and Right: EPL-catechol hydrogels (Gel 1; 0.3:0.1, Gel 2; 0.4:0.1 and Gel 3; 0.5:0.1 mmol/L), against MRAB and MRSA incubated at 37°C for 24 h.

The antimicrobial activity of EPL-catechol hydrogels was determined by the KB test (zone of inhibition) and surface contact assay. All three types of hydrogels showed promising antimicrobial activity against both MRAB and MRSA. The zone of inhibition of various EPL-catechol hydrogels against MRAB and MRSA is shown in Figure 2A,B. Generally, the inhibitory zone for MRAB is slightly larger than MRSA. The halos increase in size as the concentration of EPL increases in hydrogels. Gel 3 has the most potent antimicrobial activity and therefore, used in further experimentation.

**Figure 3:**
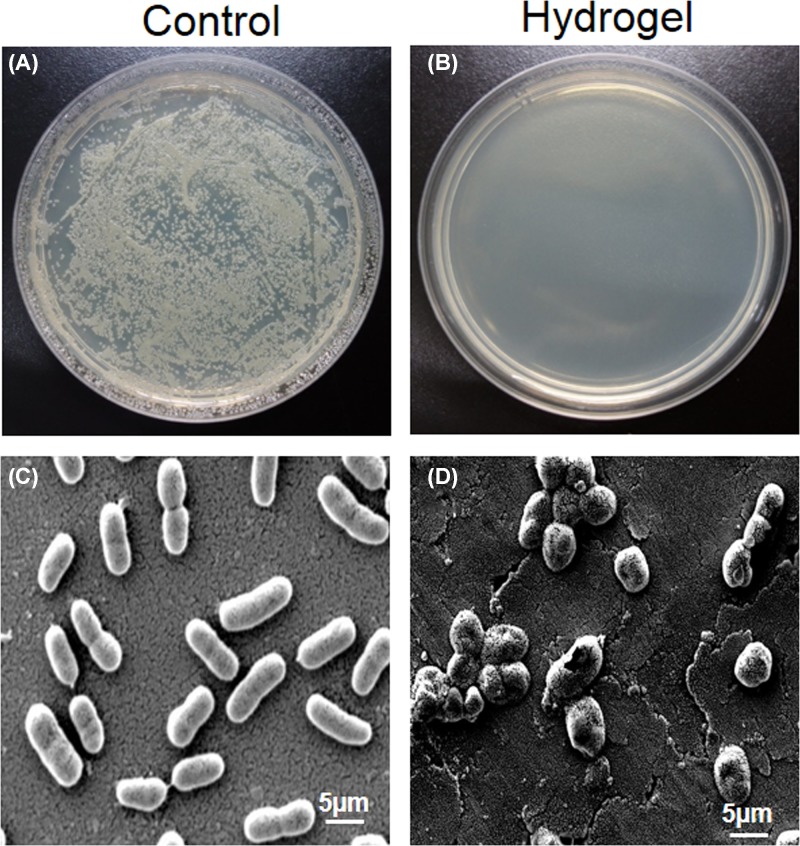
*In vitro* antimicrobial activity of EPL-catechol hydrogel against MRAB on surface contact Top; (**A**) MRAB colonies grown on agar plate after contact with control (PEGDA hydrogel) and (**B**) EPL-catechol hydrogel for 2 h. Bottom; SEM images representing MRAB contact with (**C**) control (PEGDA hydrogel) and (**D**) EPL-catechol hydrogel for 2 h.

The antimicrobial activity of the hydrogel was further evaluated by adding an MRAB suspension on to the surface of the EPL-catechol hydrogel (Gel 3) at a concentration of 1 × 10^8^ CFU/cm^2^. PEGDA hydrogel was used as a control. Interestingly, following 24 h of incubation, the EPL-catechol hydrogel showed an almost 100% killing of MRAB compared with the control hydrogel (Figure 3). A large number of MRAB colonies were observed after contact with the control PEGDA hydrogel (Figure 3A). However, no viable MRAB colony was detected after contact with the EPL-catechol hydrogel (Figure 3B). To further verify this phenomenon, the hydrogel samples carrying bacteria were observed under SEM. MRAB was seeded on the surface hydrogels for 2 h and then prepared for observation using SEM. Marked structural alterations were observed in the cell morphology of MRAB in contact with EPL-catechol hydrogel compared with the control PEGDA hydrogel. The bacterial cells of the control group (Figure 3C) exhibited rounded, rod-like and smooth appearances, whereas the bacteria in contact with EPL-catechol hydrogel showed wrinkled, disrupted and withered surfaces (Figure 3D).

**Figure 4:**
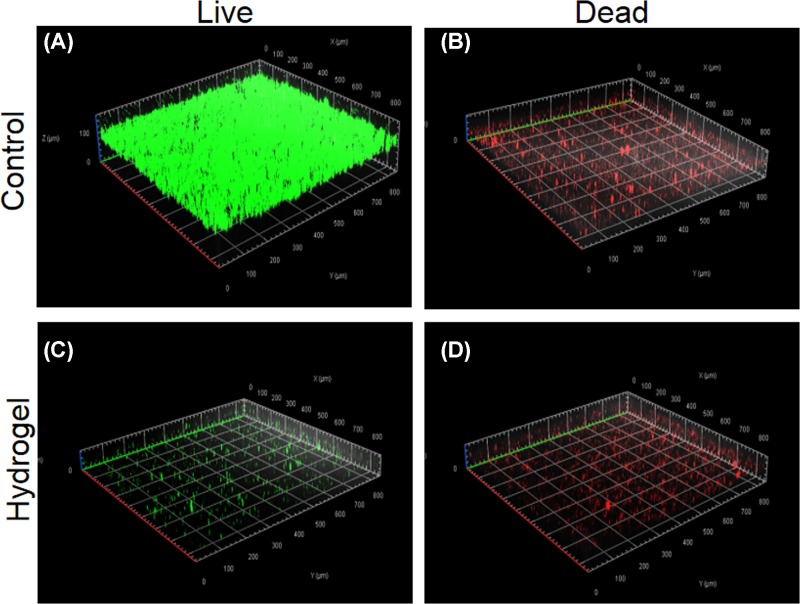
Antibiofilm action of EPL-catechol hydrogel Confocal fluorescence microscopic images of LIVE/DEAD Backlight bacterial viability kit stained MRAB on the surface of (**A,B**) TCPS control and (**C,D**) EPL-catechol hydrogel after 3 days of growth.

The antibiofilm activity of EPL-catechol hydrogel was also studied using Gel 3. A LIVE/DEAD bacterial cell viability assay was carried out for the visualization of MRAB biofilm formation on the surface of the EPL-catechol hydrogel and control TCPS. The bacterial viability was carried out to confirm the bactericidal effect of our EPL-catechol hydrogel. LIVE cells were stained fluorescent green, while DEAD cells with damaged membrane were stained red [35]. in our studies, MRAB that appeared red were dead, while the green appearance indicated live bacteria. The strong green fluorescence signals shown in Figure 4A,B indicate that a biofilm containing many bacteria were grown on the TCPS (control) after 3 days of incubation, and most of the MRAB were alive. In contrast, only a few sporadic live/dead bacteria cells were observed on top of the EPL-catechol hydrogel (Figure 4C,D). A significant reduction in MRAB survivability was obtained from the EPL-catechol hydrogel compared with the TCPS (control). These results clearly indicate that bacterial cells adhere to TCPS and proliferate well during incubation and form a biofilm, whereas the EPL-catechol hydrogel successfully inhibited biofilm formation.

### *In vitro* biocompatibility of EPL-catechol hydrogel

**Figure 5:**
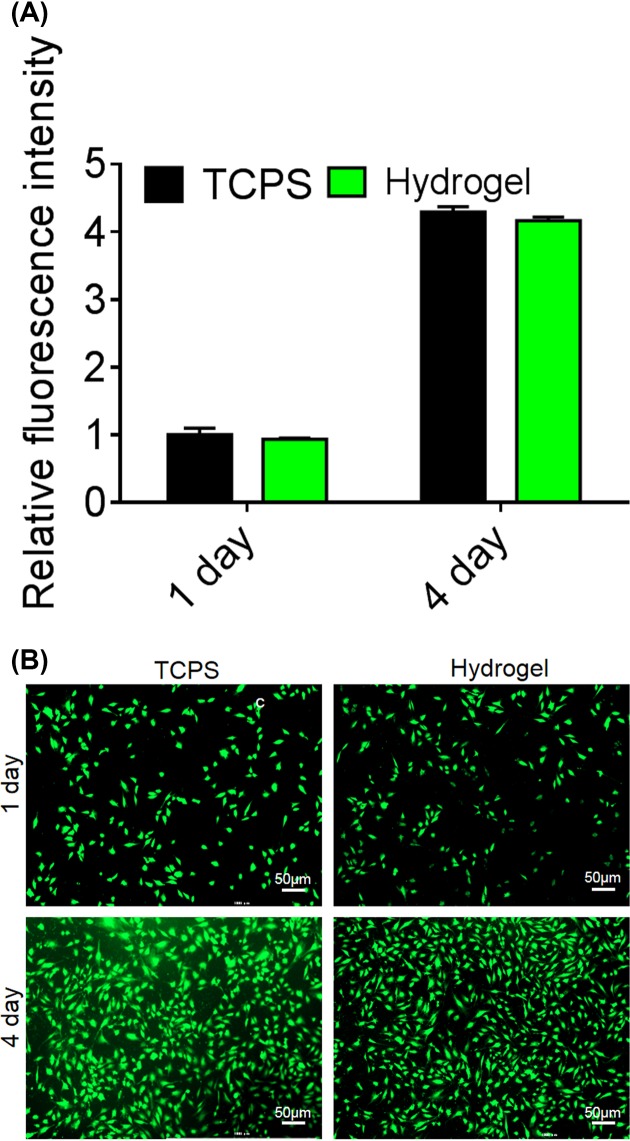
*In vitro* biocompatibility of EPL-catechol hydrogel (**A**) Relative fluorescent intensity and (**B**) LIVE/DEAD fluorescent images of C2C12 cells, cultured in the presence of EPL-catechol hydrogel and TCPS (control) for 1 and 4 days.

The cytotoxicity of EPL-catechol hydrogel (Gel 3) was assessed with the C2C12 cell line by performing an alamarBlue assay. The hydrogel was directly placed on cells and cultured for 1 and 4 days. As shown in Figure 5, EPL-catechol led to slightly reduced cell viability on days 1 and 4 compared with the control TPCS group. However, there was no statistical significant difference between the control group and the EPL-catechol hydrogel. The absorbance of the cells in contact with the EPL-catechol hydrogel increased with culture time, thus indicating periodic cell growth. The cells proliferated well in the presence of hydrogel, indicating the good biocompatibility of the EPL-catechol hydrogel.

### *In vivo* antimicrobial activity of EPL-catechol hydrogel

**Figure 6:**
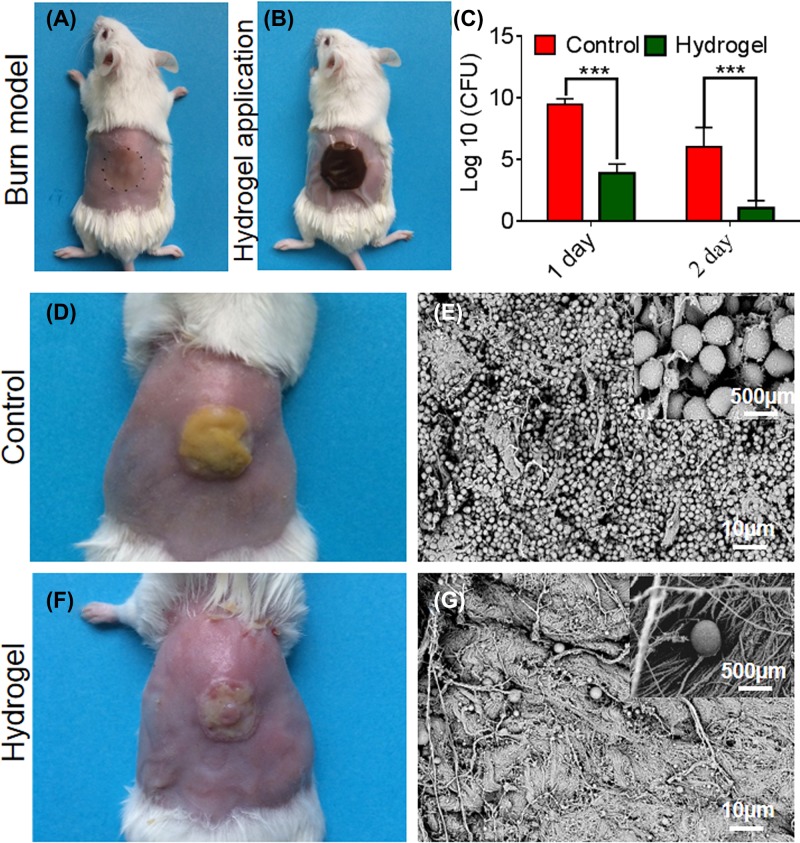
*In vivo* antimicrobial activity of EPL-catechol hydrogel (**A**) The burn wound on mice dorsal skin loaded with 1 × 10^8^ CFU/ml of MRAB, (**B**) then covered with EPL-catechol hydrogel. (**C**) The bacterial CFU on the mice burn wound on days 1 and 2. Data are expressed as the mean ± standard deviation (*n* = 6) (*P* < 0.001). (**D**) MRAB biofilm formation (**E**) SEM images of control group (PEGDA hydrogel) and (**F**) biofilm disruption (**G**) SEM images after treatment with EPL-catechol hydrogel for 2 days.

The *in vivo* antimicrobial activity of EPL-catechol hydrogel against MRAB was determined by measuring MRAB burden in burn wound (Figure 6). The burn wound (Figure 6A) inoculated with MRAB followed by EPL-catchol hydrogel application (Figure 6B) showed that the number of MRAB in the burn wound reached 1.2 × 10^9^ CFU/g after 24 h in the control group (PEGDA hydrogel). However, MRAB colonies under the EPL-catechol hydrogel dressing were found to be 2.1 × 10^4^ CFU/g, showing a log reduction of 4.76 from the control group. After 48 h, although the number of MRAB colonies in the control group decreased to 1.1 × 10^7^ CFU/g, the number of colonies in burn wounds treated with EPL-catechol hydrogel was further reduced to an average of 22 CFU/g. The log reduction between them is 5.70. The application of EPL-catechol hydrogel significantly reduced the bacterial burden on days 1 and 2 of treatment compared with the control group (Figure 6C, *P*<0.001).

Upon observing the wound appearance, the control group showed yellow slough due to severe infection (Figure 6D), while the EPL-catechol hydrogel-treated burn wound had improved appearance (Figure 6F). SEM observations were carried out to observe the bacterial growth in the burn wounds. Fewer bacteria were observed on the burn wound treated with EPL-catechol hydrogel (Figure 6G) compared with the control group (PEGDA hydrogel), where MRAB proliferated in high numbers, leading to biofilm formation (Figure 6E). The EPL-catechol hydrogel prevented biofilm formation.

### *In vivo* biocompatibility of EPL-catechol hydrogel

**Figure 7:**
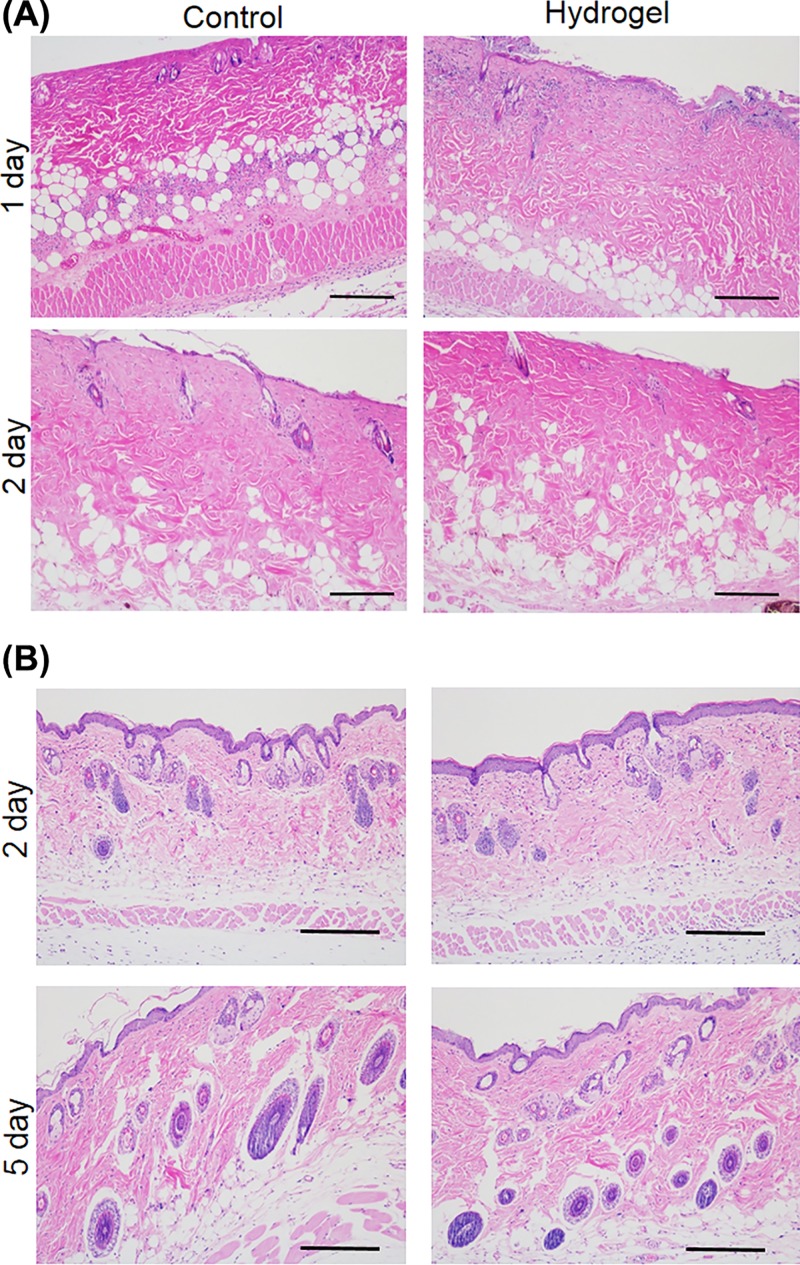
*In vivo* biocompatibility study of the EPL-catechol hydrogel (**A**) Histological analysis of mice dorsal skin after 1 and 2 days of control group (PEGDA hydrogel) and EPL-catechol hydrogel application. (**B**) H&E of skin tissue samples after 2 and 5 days of the subcutaneous EPL-catechol hydrogel implantation. Original magnification 100×, Scale bar: 100 µm.

The *in vivo* tissue toxic effect was evaluated by topically applying EPL-catechol hydrogel (Gel 3) for 1 and 2 days and a subcutaneous implant for 2 and 5 days (Figure 7). We observed histological alterations via H&E staining of the surrounding tissue sections. Notably, the EPL-catechol hydrogel showed no inflammatory cells infiltration on day 1 and 2 of topical application compared with the untreated control group (Figure 7A). Likewise, the subcutaneous EPL-catechol hydrogel implantation showed no signs of inflammation and displayed the normal architecture of epidermis, dermis, and subcutaneous tissues similar to the untreated tissue samples on day 2 and 5 of implantation (Figure 7B and Supplementary Figure S2B,C). These results are consistent with the EPL-catechol hydrogel promising biocompatibility.

## Discussion

Burn patients are at high risk of infection as a result of the nature and extent of burn injuries and prolonged hospital stays. Burn injury compromises skin integrity and immunity, affecting crucial functions such as homeostasis and protection against infection [1]. Hydrogel wound dressings were shown to contribute to wound debridement by the rehydration of nonviable tissue [36], necessary for wound healing. However, current hydrogel wound dressings cannot effectively control microbial growth. In this work, we presented a catechol cross-linked EPL hydrogel with potential antimicrobial action against multidrug-resistant microbes such as MRSA and MRAB.

The EPL-catechol hydrogels were prepared by *in situ* cross-linking of the amine groups of EPL molecules with catechol in a chemical reaction (Scheme 1) [37,38]. During the reaction process, the generation of *o*-quinone by oxidative conversion of the catechol moiety changes the color into brown [39]. As shown in Scheme 1, the EPL-catechol hydrogel turns brown, suggesting the occurrence of reaction [39]. The SEM reveals the homogeneous microporous structure of the hydrogel. The hydrogel structure became more porous as the concentration of EPL increased (Figure 1). The increasing EPL concentration of hydrogel also results in an increase in preserving water content above 90%, which could provide a moist environment for faster wound healing. The adjustable EPL plays an important role in the porous structure and water content of hydrogels, which can meet different clinical demands.

EPL is valued as a safe, thermostable and most importantly a strong antimicrobial agent. It has been widely used for food preservation and has been approved by the U.S.A., Korean and Japanese administrations [40]. EPL has a broad-spectrum antimicrobial action against both Gram-negative and Gram-positive bacteria [17]. In our *in vitro* studies, the EPL-catechol hydrogel showed potent antimicrobial effects against clinical MRAB and MRSA bacterial strains. An increase in bacterial inhibition was noticed with the increase in EPL concentration in various hydrogels (Figure 2).

SEM studies showed that MRAB contacting the EPL-catechol hydrogel surface led to cell wall rupture and surface roughness of bacteria indicating the disruption of the bacterial cell membrane (Figure 3). Furthermore, MRAB cells were alive and growing well on TCPS (control) but dead and inhibited on the EPL-catechol hydrogel. This mechanism is consistent with the findings that EPL kills bacteria by adsorbing on to the cell surface and perturbing the cell outer membrane, leading to the abnormal distribution of the cytoplasm and ultimate damage to the microbe [41]. EPL has a positively charged hydrophobic segment of –(CH_2_)_4_– in each repeating unit of the backbone, interacts with the pathogen’s anionic and hydrophobic membrane surface [17]. Thus, SEM and LIVE/DEAD assays collectively suggest that the EPL-catechol hydrogel is effective against MRAB by damaging the bacterial cell membrane (Figure 4). These results are consistent with previous reports showing that EPL distributes a positive charge and results in cell membrane fracture [29,42]. It is crucial for functionalized hydrogels to possess biocompatibility, which is important for wound healing [43]. Our results showed that the EPL-catechol hydrogel is nontoxic to C2C12 cells and possesses excellent cytocompatibility (Figure 5).

Recently, bioluminescent models are used widely to analyze the antimicrobial activity of drugs and evaluate bacterial resistance to antimicrobial agents [44,45]. Bioluminescent *S. aureus* models have been studied to assess its pathogenesis in deeper host tissues and for drug efficacy evaluation [46]. Bioluminescent models of bacterial infection and wound sepsis are interesting research approach toward accurate representation of different stages of infection and biofilm formation. Ogunniyi et al. [47], successfully reported bioluminescent *S. aureus* infection and treatment in partial thickness and second-degree burn. The use of bioluminescent *A. baumannii* and *S. aureus*, may provide reliable future models to assess preclinical efficacy of EPL-catechol hydrogel in treating burn wounds infection. However, bioluminescent *A. baumannii* resistance toward various antimicrobial drugs is yet to be evaluated. In our study we used clinical strain of *A. baumannii* which has shown resistance to multiple drugs (Supplementary Table S1).

Burn wound infection caused by MRAB is a global healthcare issue due to its inherent antibiotic resistance. MRAB have become resistant to carbapenems and to last-resort antibiotics such as colistin and tigecycline [48]. To demonstrate the clinical potential of our designed EPL-catechol hydrogel, the MRAB-infected burn wound was treated with hydrogel for 2 days and examined by SEM. We observed fewer bacteria cells in the EPL-catecol hydrogel-treated burn wound compared with the control group, where a large number of bacteria adhered to the wound bed (Figure 6). These results clearly demonstrated the *in vivo* antimicrobial action of EPL-catechol hydrogel.

It is important to prepare a hydrogel that nontoxic toward mammalian cells. The subcutaneous implantation of the hydrogel showed no signs of inflammation and displayed normal architecture of the epidermis, dermis and subcutaneous tissues, similar to the untreated control group (Figure 7). Thus, the EPL-catechol hydrogel is a promising wound dressing material.

## Conclusion

In the present study, for the first time, we established a novel interlaced microporous EPL cross-linked with catechol hydrogel**.** We demonstrated that EPL-catechol hydrogel not only exhibits antimicrobial but also antibiofilm activity toward multidrug-resistant bacteria. We noticed that the EPL-catechol hydrogel is biocompatible and noncytotoxic to mammalian cells. The *in vivo* study indicated a significant reduction in more than four orders of magnitude of the bacterial burden in contaminated burn wounds. The histological examination of mice skin upon hydrogel application did not exhibit any signs of inflammation or tissue alteration. These results provide the basis of EPL-catechol hydrogel use for treating contaminated burn wounds and biofilm-related infections. However, further studies needs to be conducted to evaluate the hydrogel clinical efficacy.

## Supporting information

**Supplementary Figure S1:** 

**Supplementary Figure S2:** 

**Supplementary Figure S3:** 

**Supplemental Table S1:** 

## Abbreviations

AMP: antimicrobial peptide
ATCC: American type culture collection
DMEM: Dulbecco’s modified Eagle’s solution
EPL: ε-poly-l-lysine
FBS: fetal bovine serum
H&E: Hematoxylin and Eosin
MH: Mueller-Hinton
MRAB: multidrug-resistant *Acinetobacter baumannii*
MRSA: methicillin-resistant *Staphylococcus aureus*
OD: optical density
PBS: phosphate buffered saline
PEGDA: polyethylene glycol diacrylate
SEM: scanning electron microscopy
TCPS: tissue culture polystyrene
TSB: tryptic soy broth
