# Supplementary material for: Catechol cross-linked antimicrobial peptide hydrogels prevent multidrug-resistant Acinetobacter baumannii infection in burn wounds
Source: Biosci Rep. 2019 Jun 18;39(6):BSR20190504. doi: 10.1042/BSR20190504 (PMC6579981; doi:10.1042/BSR20190504)
Supplement: Supplementary file 1 [file bsr20190504_Supp1.pdf]

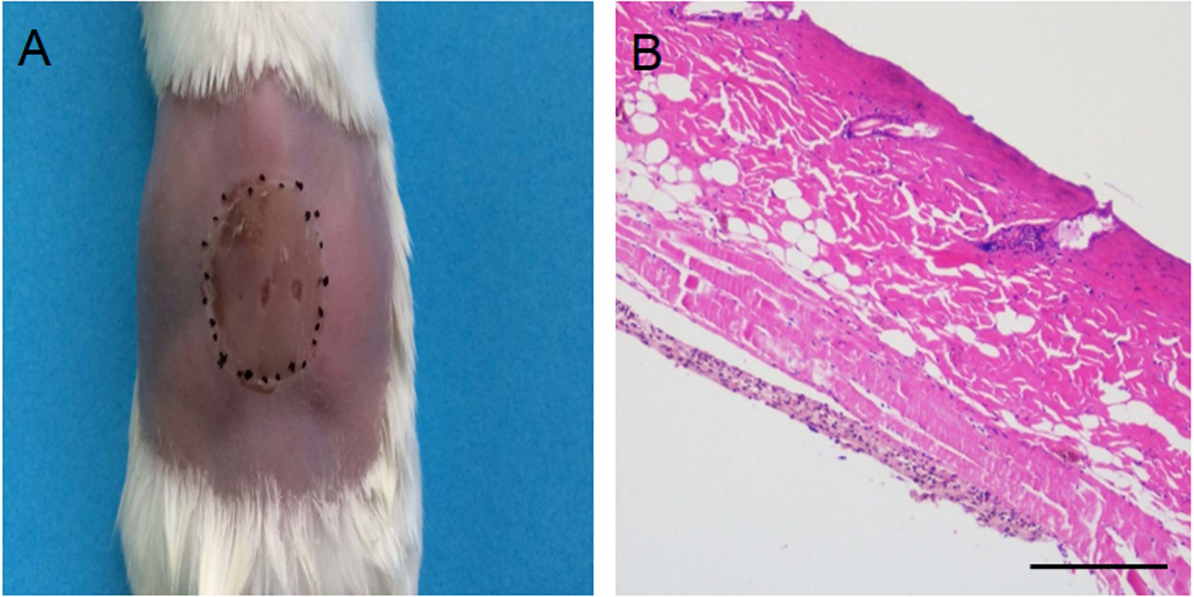
A
B

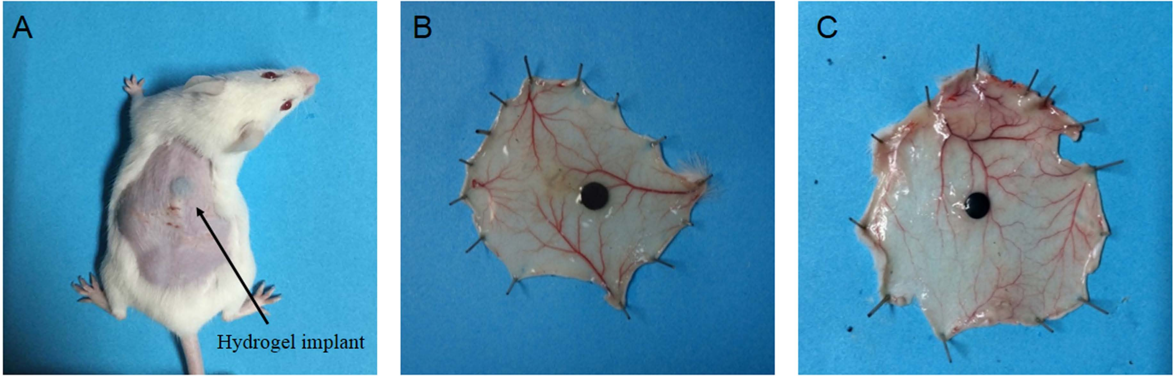
A
Hydrogel implant
B
C

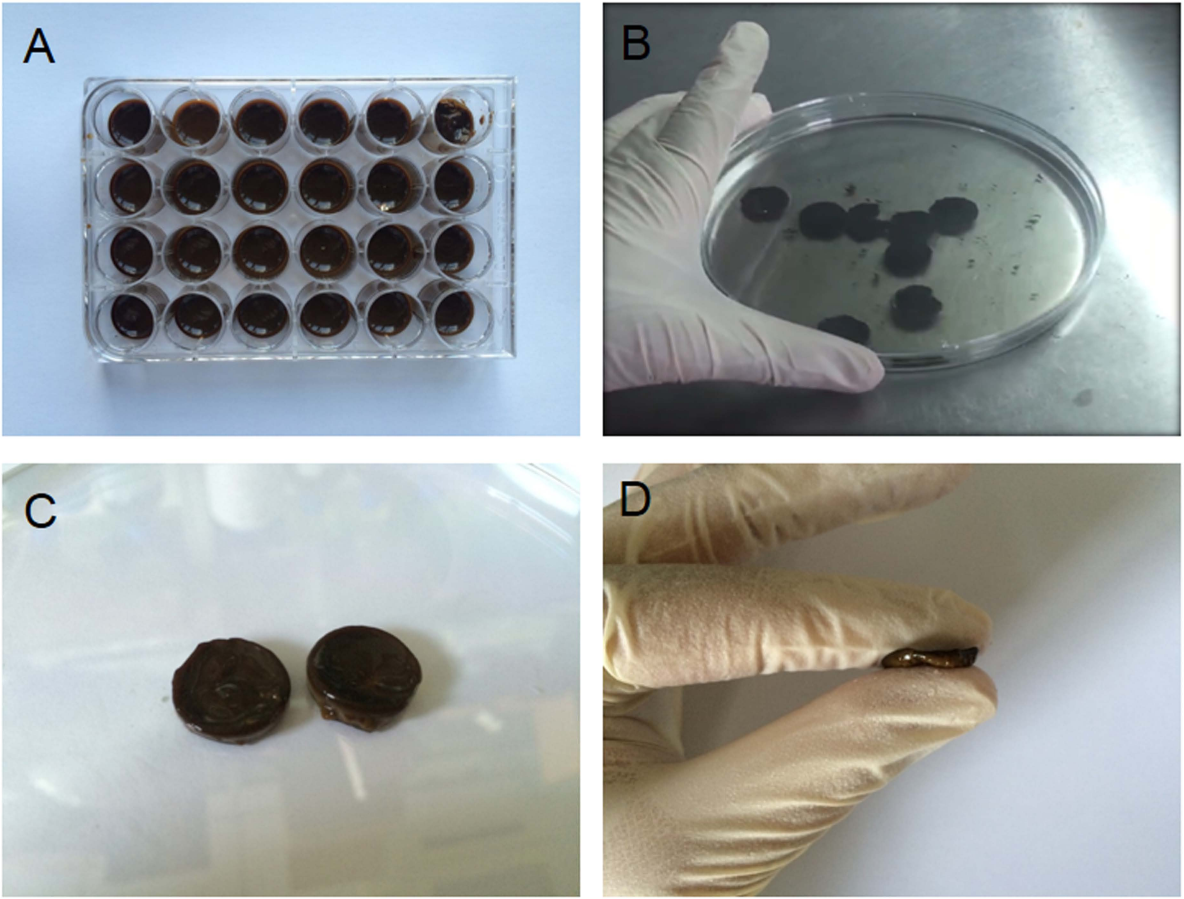
A
B
C
D

Antibiotics	Susceptibility
Ciprofloxacin	R
Levofloxacin	I
Ceftriaxone	R
Cefoperazone + Sulbactam	S
Gentamicin	R
Meropenem	R
Tigecycline	S
Tobramycin	R
Compound trimethoprim	R
Trimethoprim	R
